# SAW RFID-Tags for Mass-Sensitive Detection of Humidity and Vapors

**DOI:** 10.3390/s91209805

**Published:** 2009-12-03

**Authors:** Peter A. Lieberzeit, Christian Palfinger, Franz L. Dickert, Gerhard Fischerauer

**Affiliations:** 1 Department of Analytical Chemistry and Food Chemistry, University of Vienna, Waehringer Strasse 38, A-1090 Vienna, Austria; E-Mails: Peter.Lieberzeit@univie.ac.at (P.A.L.); Christian.Palfinger@univie.ac.at (C.P.); 2 Chair of Measuring Technique and Control Engineering, Bayreuth University, Universitätsstraβe 30, D-95440 Bayreuth, Germany; E-Mail: Gerhard.Fischerauer@uni-bayreuth.de

**Keywords:** SAW, RFID-Tag, calix[n]arene, humidity, VOC sensing

## Abstract

One-port surface acoustic wave (SAW) devices with defined reflector patterns give characteristic signal patterns in the time domain making them identifiable and leading to so-called RFID-Tags. Each sensor responds with a burst of signals, their timed positions giving the identification code, while the amplitudes can be related to the analyte concentration. This paper presents the first combination of such a transducer with chemically sensitive layer materials. These include crosslinked polyvinyl alcohol for determining relative humidity and *tert*-butylcalix[4]arene for detecting solvent vapors coated on the free space between the reflectors. In going from the time domain to the frequency domain by Fourier transformation, changes in frequency and phase lead to sensor responses. Hence, it is possible to measure the concentration of tetrachloroethene in air down to 50 ppm, as well as 1% changes in relative humidity.

## 1. Introduction

Surface acoustic wave (SAW) [1] devices have already been successfully applied as sensors in analytical chemistry [2,3], because their high resonance frequency leads to excellent sensitivity [4-7]. The concept can be developed further by including means to distinguish different devices in multisensory environments. So-called SAW-based RFID-Tags are only one out of several technological ways to realize this concept for chemical sensing applications. Others include the design of sensor arrays either with sequential read-out or multiplexing. In contrast to these, RFID tags in principle allow for interrogating all sensors simultaneously, followed by deconvolution of the resulting signal.

Common two-port SAW devices usually consist of a piezoelectric substrate (e.g., ST-cut quartz) having two metallic interdigital transducers (IDT) deposited on its surface. Applying an electrical signal to one of the IDT triggers a mechanical acoustic wave on the surface that is re-transformed into an AC signal on the second transducer. In contrast to this, RFID-Tags consist of SAW with only one IDT and a distinct reflector pattern [8] leading to time-dependent signal modulation that is suitable for identifying individual devices. Generally, the resonance frequency of surface acoustic wave devices is determined by the structure width of the IDT. In the same way as standard SAW, RFID-Tags can be coated with a sensitive layer to achieve chemical sensors. Such a layer modifies the velocity of the surface acoustic wave resulting in frequency shifts [9]. The underlying physical processes leading to these frequency changes in SAW, however, are still not fully elucidated. Anyway, for an ideal thin film with low elastic modulus, they shift can be calculated as follows:

(1) $$\Delta f=(k_{1}+k_{2})f_{0}^{2}V_{F}\rho -k_{2}f_{0}^{2}t_{F}\frac{4\mu \cdot (\chi +\mu )}{v_{r}^{2}(\chi +2\mu )}$$

where, k_1_ and k_2_ are material constants of the SAW device, f_0_ is the fundamental frequency, V_F_ the film volume, ρ its density, μ the elastic modulus, χ the Lamé constant and V_r_ the Raleigh velocity of the film, respectively. Normally, the interaction between film and sensor does not change the elastic properties of the former. Hence, the second term in Equation1 can be neglected yielding the following relationship giving linear correlation between mass change and frequency shift:

(2) $$\Delta f=k_{1}f_{0}^{2}t_{F}\rho =k_{1}f_{0}^{2}\Delta m$$

Analyte incorporation into the layer then generates further frequency shifts, which are recorded and yield the desired sensor responses. In addition to the functionality of a SAW device, IDT in principle also allow for implementing wireless interrogation into a sensor system: by connecting an antenna to the interdigital electrodes, the element can be used as passive system without the need of on-board power supply [10]. Such devices are e.g., already used for wireless measurement of physical data and are industrially implemented in a road pricing system in Norway. To the best of our knowledge, we report the first combination of such a device and a chemically selective layer material. Currently, RFID-Tag sensors proposed in literature usually focus on physical sensing or detect chemical changes rather directly with the device than with a rationally designed recognition material. Our aim here thus is to deliver experimental proof of concept of RFID-Tags as chemical sensors. For applying them as sensors in real-life environments at a later stage, it will of course be necessary to address questions of packaging, control units and installation.

**Figure 1.**
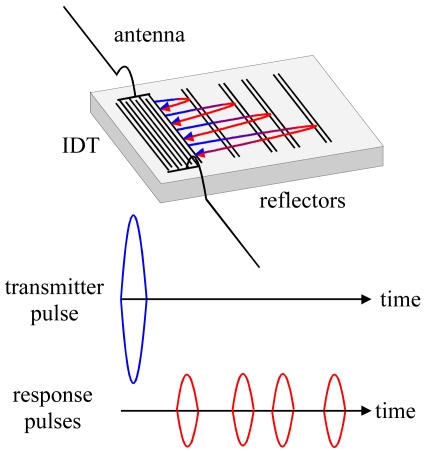
RFID-Tag with interrogation and response signals.

**Figure 2.**
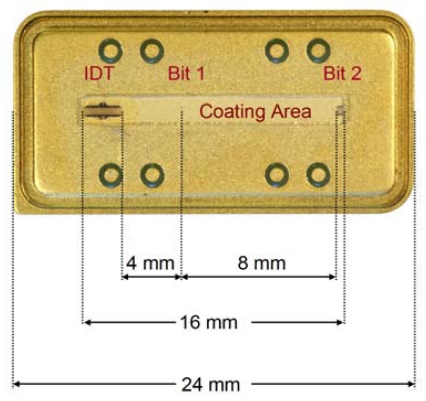
RFID-Tags used for chemical sensing.

Figure 1 shows the structure of an RFID-Tag. When it is interrogated by a radio frequency signal (be it locally or remotely), the sensor responds with a burst of pulses depending on the number and position of reflectors, yielding an identification code. For chemical sensing purposes, it is necessary to apply transducers offering sufficient space for depositing a recognition material. Therefore, we used the geometrical setup depicted in Figure 2. In such a system, the sensitive layer incorporates the species of interest, which changes the attenuation of the signals leading to quantitative information after analysis. Sensor responses can be obtained both from measuring frequency and phase changes, respectively. Within this work, the fundamental frequency of the devices equals 434 MHz, which is a very typical value in sensor applications. As proof of concept for chemical sensing, we thus coated RFID tags with optimized selective layers: hydrophilic polymers for water detection and molecular cavities for an example of an organic solvent molecule lacking pronounced functionalities, namely tetrachloroethene. Supramolecular recognition based on such molecular cavities has already proven to be highly suitable for detecting volatile organics [11].

## 2. Results and Discussion

### 2.1. Instrumental Aspects

**Figure 3.**
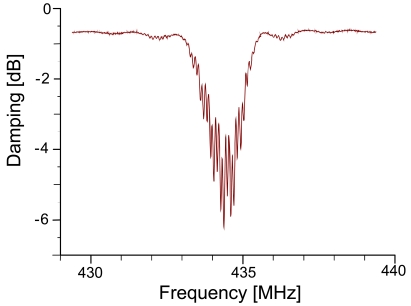
Sensor response in frequency domain of an RFID-Tag.

Figure 3 shows the response of the RFID-TAG in the frequency domain: the different reflector electrodes lead to overlapping waves and thus lead to an interference pattern. The bandwidth of this signal depends on the number of reflecting “Bits” (*i.e.*, reflectors) deposited on the transducer.

**Figure 4.**
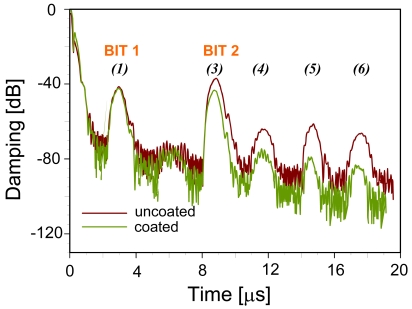
Sensor response in time domain—identification code of the uncoated (red line) and the PVA-coated (green line) RFID-Tags, respectively.

**Figure 5.**
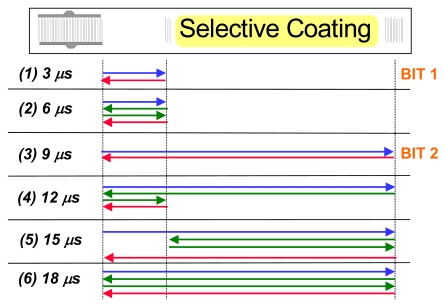
Surface wave reflections leading to the response pattern in Figure 5.

Fourier-transformation from the frequency domain into the time domain leads to the pattern depicted in Figure 4, shown for both a coated and an uncoated device. The two reflectors give rise to the main signals at approximately 3 and 9 μs, respectively. The other minor peaks are generated by multiple reflections between the reflectors and the IDT. Figure 5 summarizes these reflections on the device surface explaining the above signals.

There, blue and red arrows denote the signal emitted and re-transformed by the IDT, respectively (these colors correspond to the ones used in Figure 1). Green arrows symbolize surface waves reflected between different bits and the IDT, respectively, without generating an electronic signal.

Additionally, Figure 4 also gives evidence on the influence of the sensitive layer: when comparing the signals of the coated and the uncoated device, respectively, one can see that the sensitive layer damps all signals having been reflected at least once by Bit 2. Therefore, also changes of the layer during sensing events can be expected to result in sensor responses. Device geometry also supports this: RFID-tags have much longer propagation pathways as compared to surface acoustic wave resonators (8 mm and 50 μm, respectively) thus substantially increasing the influence of the recognition material on the electronic behavior. As a consequence, coatings can be kept very thin in order to reduce response times substantially leading to optimized systems for on-line monitoring and surveillance.

The devices applied in this study use quartz as the piezoelectric material for transforming the electrical signal into a mechanical one and vice versa. It is well known that such devices show parabolic relationship between temperature and frequency response with the apex at room temperature. Given that this study has looked for a proof of concept for combining RFID tags with selective recognition materials as well as the application in mind (*i.e.*, indoor monitoring), temperature thus only plays minor role, as it can be expected to vary by not more than ±20 °C. In this region, hysteresis also does not play a role.

**Figure 6.**
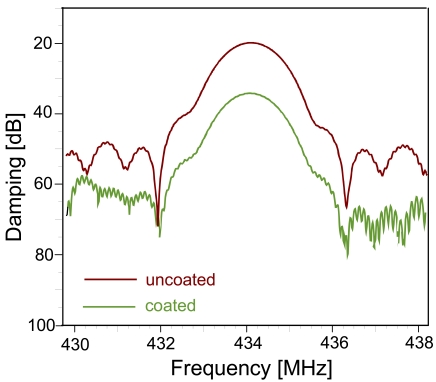
Frequency spectra of the 9 μs signal of the uncoated and the coated RFID-Tag sensor response in time domain.

For quantitative sensing we extracted the signal around 9 μs and re-transformed it into the frequency domain (see Figure 6) thus eliminating interferences. On this signal, we performed all measurements using the −3 dB point, which is defined by the frequency, which is damped by −3 dB more than the resonance maximum. However, when collecting the data at constant damping, frequency shifts can be observed. Therefore, we monitored both the phase shifts at a defined frequency (434 MHz) and the frequency shifts at a distinct attenuation as a function of humidity or organic vapor concentration in air.

To record the sensor responses, we placed both uncoated and coated devices into a stream of air containing exactly defined amounts of water or tetrachloroethene vapor, respectively. These, we generated by mass flow controllers (Tylan FC 260) and validated with an FT-IR gas cell (Perkin Elmer System 2000, gas cell with path length from 7.5 m, Siege Company, Foxboro® LV7).

### 2.2. Humidity Sensor

**Figure 7.**
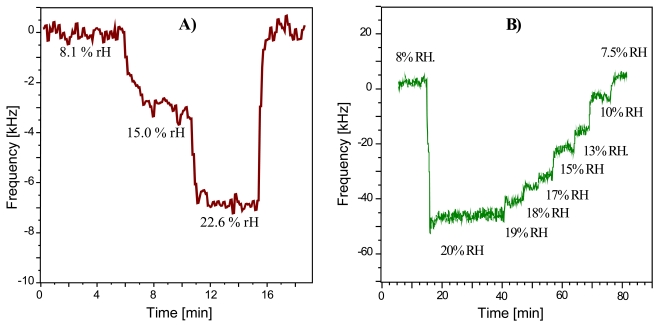
(a) Sensor response (frequency shifts at a distinct attenuation) of the uncoated RFID-Tag to varying amounts of humidity (T = 20 °C, flow: 1 L/min); (b) Sensor response (frequency shifts at a distinct attenuation) of the PVA coated RFID-Tag to varying amounts of humidity.

The resonance frequency of the RFID-Tag response at 9 μs is placed at approximately 434 MHz and shows a bandwidth of 1.4 MHz. At first we examined the sensor response to humidity at the −3 dB point as described. Figures 7A/B depict the resulting data: the response curve in Figure 7A results from exposing an uncoated RFID-Tag to air with variable humidity. The noise level is within 1 kHz and the sensor response to 20% rH is 7 kHz. These frequency shifts are a consequence of the hydrophilic properties of the SAW quartz substrate. Compared to these shifts, the coated devices lead to substantially higher frequency responses in the same humidity range, as is can be seen in Figure 7B. The noise level is about 7 kHz and the sensor response to 20% rH nearly 50 kHz. Evidently, the coated device reacts much more sensitively to being exposed to humidity. These findings show the highly appreciable performance of the coating material used, since applying a PVA layer increases the sensor response by nearly a factor of ten. It should also be noted that the data depicted in Figure 7 has been obtained without any noise reduction. By applying e.g., averaging over some data points, it is possible to distinguish humidity differences being as small as 1% rH even at low water vapor concentrations in air. Such polymer-based affinity sensors are especially interesting the humidity range below 20% rH, because higher levels are already straightforwardly accessible by uncoated lithium niobate SAW (or RFID-Tag) devices (however, they fail at lower rH). The RFID-Tag sensor does not only exhibit excellent reversibility but also appreciably short response times amounting to a few minutes, limited only by the gas-mixing apparatus used. Furthermore, the sensor characteristics obtained indicate linear calibration function at a low humidity. When calculating the difference in sensor response between coated and uncoated device, linear regression yields a calibration function with 3.35 kHz/%rH slope and 0.9936 regression factor. Furthermore, this layer also shows excellent long term stability, as it was possible to verify these results in consecutive measurements after 2 years. One might argue that the effects caused by the sensing events are in the same frequency range as the variability between different devices caused by slight variations during the production procedures. However, the sensor signal evaluated always is the frequency shift between caused by analyte exposure. Therefore, the resonance frequency of the device is always set on zero value thus compensating for the variability of the RFID-tags. Taking into consideration the sensor characteristics, one can see sensitivity being slightly below 10 kHz per percent relative humidity which in terms of absolute humidity at 20 °C means 1 kHz/20 ppm. Given the low molar mass of water, this strongly supports the sensitivity of the devices.

### 2.3. Tetrachloroethylene Sensor

**Figure 8.**
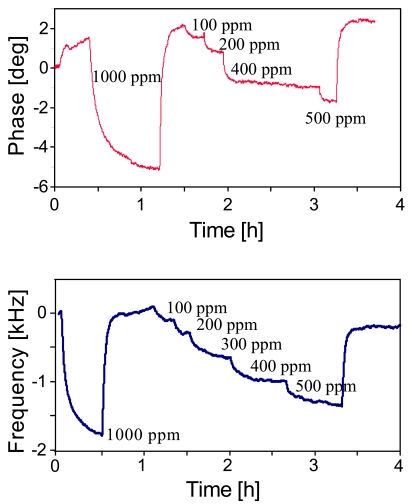
(A) Sensor response (phase shift at a distinct frequency) of tert.-butylcalix[4]arene coated RFID-Tag to various amounts of tetrachloroethylene (T = 20 °C, flow: 1 L/min), (B) corresponding frequency shifts.

Whereas humidity is accessible by comparably straightforward affinity layers, selectively detecting organic solvent vapors requires rationally designed recognition sites, such as e.g., supramolecular cavities. After coating the respective IDT with calix[4]arene, we again monitored the phase shifts at a fixed frequency (434 MHz, see Figure 5) yielding the sensor responses shown in Figure 8A. The phase shift resulting from exposure to 500 ppm tetrachloroethylene is about 4° with linear sensor characteristic and lower detection limit of approximately 50 ppm thus meeting the IUPAC requirements (*i.e.*, three times the standard deviation of the noise is the smallest detectable signal) for reliably determining the maximum workplace concentration of tetrachloroethylene. All measurements indicate that the sensors react fully reversibly with response times are within some minutes. However, the latter is mainly a consequence of the gas mixing apparatus, as sensitive layers for SAW are usually some ten nm thick leading to response within seconds. Sensor results can be further improved by recording the frequency shifts as a function of analyte concentration rather than the phase angle, as can be seen from the data presented in Figure 8B. The response of the coated device is ten times higher than the one of the uncoated, while the noise level remains the same. Sensor layers in this case are in the range of about 100 nm. With this setup we thus successfully achieved a detection limit of less than 50 ppm according to IUPAC standards. Measurements with unusually high concentration (1,000 ppm, not shown) indicate saturation of the sensor responses, however, in the analytically relevant range the sensors exhibit linear sensor characteristic with slope of 3.49 Hz/ppm and R^2^ = 0.9901. Comparing the results for both water and tetrachloroethene with previous measurements done with standard SAW devices of the same fundamental frequency (results not shown) indicates that the RFID tags have similar sensitivity, *i.e.*, around 1.5 Hz/pg frequency response. Furthermore, sensor signals reach their initial values after turning off analyte exposure, which strongly indicates that no hysteretic effects occur at least for chemical applications.

## 3. Experimental

### 3.1. Devices and Measurements

The RFID-Tags (Siemens AG, Type V058A/01) consist of an ST-cut quartz substrate mounted on an 8-pin-socket with the IDT connected to one of the pins and mass, respectively. The two reflectors are at four and twelve millimetres distance from the IDT leading to response pulses at 3 and 9 μs, respectively. For chemical sensing, we deposited the respective chemically sensitive layer onto the propagation pathway of the surface acoustic wave between the two reflectors. Coating details can be found in section “sensor materials”. For measurements, we applied a Hewlett Packard HP8572C network analyzer in reflection mode. To achieve time-dependent data (*i.e.*, the response pattern of the individual RFID tag), we applied chirp-Z Fourier transformation to the frequency range 434 ± 5 MHz and extracted the data region between 0 and 20 μs following excitation. By the means of a “gate” function, we then isolated the signal at 9 μs and retransformed it to frequency base to record the actual sensor characteristics. Readout of the data took place via HPIB/GPIB interface through custom-made software.

### 3.2. Sensor Materials

RFID-Tags for humidity sensing were coated with polyvinyl alcohol (PVA). This material provides substantial number of hydroxyl groups, which are favourable interaction sites for water vapour absorption. The resulting increase in mass can then be detected by acoustic transducers [12]. For this purpose, we mixed 10 mg polyvinyl alcohol 15,000 (Fluka) with 10 mg acrylic acid (Merck) in 25 mL dimethylformamide (DMF) and radically pre-polymerised with 1 mg AIBN (Merck) at elevated temperature. Finally, 2 microlitres of this solution were evenly distributed between the two reflectors of the RFID-Tag and dried at 50 °C overnight. This procedure yielded homogeneous, robust layers not showing any bleeding. The resulting layer heights can be calculated being in the range of about 2 nm, which is reasonable giving the length of the propagation pathway on the device.

Supramolecular chemistry approaches with tert.-butylcalix[4]arene—sometimes even linked covalently to the transducer surface [13] for improved layer stability—have proven advantageous. Calix[n]arenes form molecular cavities being suitable to engulf an analyte molecule. For detecting tetrachloroethylene, we chose a lean cavity, namely *tert*-butylcalix[4]arene [14] (Sigma-Aldrich) and prepared a solution containing 10 mg of it in 25 mL DMF. In the same way as above, we homogeneously drop-coated 2 μL of this onto the RFID tag surface and dried at 50 °C.

## 4. Conclusions

To the best of our knowledge, this paper actually demonstrates for the first time how to combine remotely interrogatable surfaces acoustic wave devices with chemically selective layers. Although this type of devices is currently not the most common one among wirelessly addressable ones, they have a fundamental advantage for the application in chemical sensing: the respective analyte compound reversibly and non-covalently reacts with the recognition material and in this way directly changes the electronic properties of the respective device. We showed the feasibility of this approach for inherently wireless sensing by the examples of quantitatively detecting low relative humidity in air and by calix[n]arene coatings incorporating volatile organic compounds.

In the former case, PVA layers have proven to exceed the moisture sensing abilities of uncoated lithium niobate structures by far: without noise reduction, they allow for selectively detecting 2% rH in air, whereas averaging techniques lead to statistically significant responses for changes as small as 1% rH, which seems highly interesting e.g., for indoor air quality monitoring in air-conditioned buildings. In the latter case, the calix[n]arene macrocycles have selectively incorporated tetrachloroethylene, a very widely used dry cleaning agent. The RFID-Tag achieves the necessary recognition abilities as well as sensitivity and does in principle can contribute to workplace monitoring at dry cleaners'. Therefore, we have in principle shown the inherent versatility of the combination of a remotely accessible device and a chemically sensitive layer. Of course, real-life application of such systems still requires substantial efforts to optimize both operating electronics and layer design.
